# Supplementary material for: Comparative multiomics analysis of cell physiological state after culture in a basket bioreactor
Source: Sci Rep. 2022 Nov 23;12:20161. doi: 10.1038/s41598-022-24687-4 (PMC9686226; doi:10.1038/s41598-022-24687-4)
Supplement: Supplementary file 1 — Supplementary Information 1. [file 41598_2022_24687_MOESM1_ESM.zip › raw data/Transcriptomic raw data/6.Enrichment/1.GO/TE1vsTC1/DOWN/TE1vsTC1.down_GObar_cc.pdf]

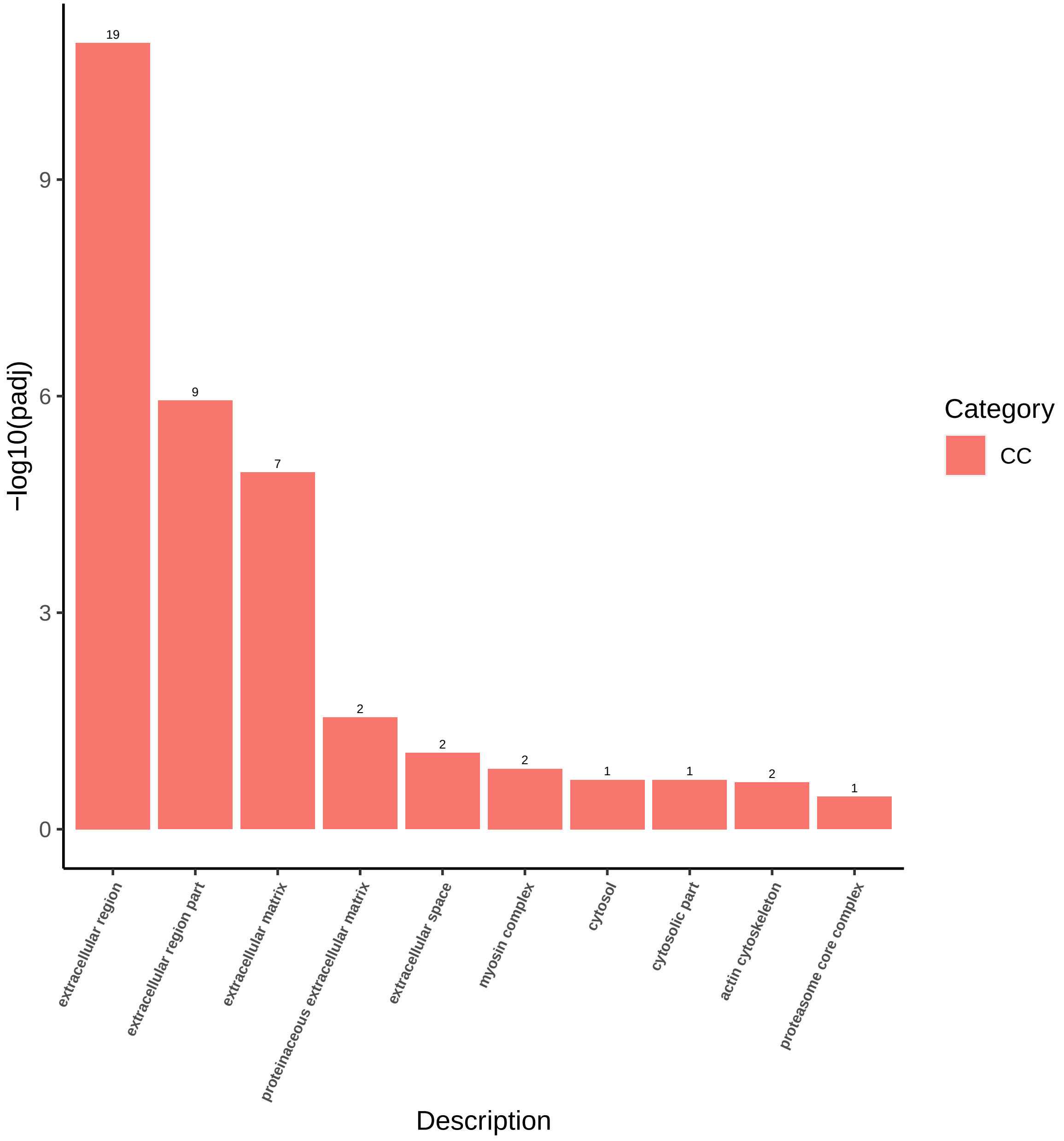

−log10(padj)
0
3
6
9
19
9
7
2
2
2
1
1
2
1
extracellular region
extracellular region part
extracellular matrix
proteinaceous extracellular matrix
extracellular space
myosin complex
cytosol
cytosolic part
actin cytoskeleton
proteasome core complex
Description
Category
CC
